# Circulating Proinflammatory Cytokines and Soluble Cytokine Receptors as Diagnostic Biomarkers in Multiple Sclerosis

**DOI:** 10.3390/jcm15062397

**Published:** 2026-03-21

**Authors:** Safia Bano, Nakhshab Choudhry, Ahsan Numan, Aamir Jamal Gondal, Nighat Yasmin

**Affiliations:** 1 Department of Neurology, King Edward Medical University, Lahore 54000, Pakistan; profahsannuman@kemu.edu.pk; 2 Department of Biochemistry, King Edward Medical University, Lahore 54000, Pakistan; drnbchoudhry@gmail.com; 3 Department of Biomedical Sciences, King Edward Medical University, Lahore 54000, Pakistan; ajgondal119@gmail.com (A.J.G.); drnyasmin@kemu.edu.pk (N.Y.)

**Keywords:** multiple sclerosis, EDSS, cytokines, interleukins, insulin-like growth factor, tumor necrosis factor, autoimmune disease, proinflammation

## Abstract

**Background**: Circulating cytokines and their soluble receptors in body fluids have been implicated in the pathogenesis of multiple sclerosis (MS). Alterations in serum levels of pro- and anti-inflammatory cytokines and/or their soluble receptors can dysregulate central nervous system (CNS) signaling pathways and, therefore, may serve as potential biomarkers for the diagnosis of MS. Therefore, the primary end-point of this study is to investigate the utility of various cytokines and their soluble receptors as diagnostic biomarkers in MS. The secondary outcome is also to assess whether these cytokines are useful in differentiating the severity of MS. **Methods**: In this case–control study, we compared a panel of pro-inflammatory interleukins (ILs), including IL18 and tumor necrosis factor-alpha (TNFα), soluble IL receptors (sIL7Rα and sIL2Rα), and insulin-like growth factor-1 (IGF-1) in 45 MS patients and in 45 healthy control individuals matched for sex and age. Associations of these biomarkers with age, disease severity (Expanded Disability Status Scale [EDSS]), disease duration, and age at first MS symptom onset were also assessed. **Results**: Serum levels of cytokines and soluble IL receptors were elevated in MS patients compared to healthy controls. IGF-1 was lower (*p* < 0.001) in the MS patients than in the healthy individuals. The serum level of IGF-1 was higher (*p* < 0.01) in the remitting-relapsing phase compared to the primary progression and secondary progression stages. Similarly, only IGF-1 was more elevated (*p* < 0.01) in the mild stage compared to the moderate stage based on the EDSS score. Receiver operating characteristic (ROC) curve analysis demonstrated that IL18 had excellent discriminatory power for the diagnosis of MS (*p* < 0.001), with an area under the curve (AUC) of 0.96 ± 0.017, followed by IGF-1 (*p* < 0.001), which showed strong diagnostic performance (AUC = 0.873 ± 0.037). Soluble (s) IL2Rα exhibited fair diagnostic accuracy (*p* < 0.001; AUC = 0.717 ± 0.054). In contrast, sIL7Rα and TNFα showed poor discriminatory power despite statistical significance (*p* < 0.01), with AUC values of 0.675 ± 0.057 and 0.687 ± 0.056, respectively. Results of regression analysis revealed that EDSS, duration of disease, and use of any treatment had no impact on the cytokines. Similarly, no significant correlations were noted between these confounders and cytokines, except a moderate negative correlation (−0.418) between IGF-1 and EDSS. **Conclusions**: IL18 and IGF-1 have the potential to be used as biomarkers in distinguishing MS from healthy individuals. However, both biomarkers failed to demonstrate the discrimination between various phenotypic patterns of disease, limiting their utility for disease stratification. Future studies with larger, longitudinal cohorts and multi-marker panels are warranted to validate these results and to explore whether combining cytokines with imaging or genetic markers can improve prognostic precision.

## 1. Introduction

Multiple sclerosis (MS) is a chronic autoimmune inflammatory disorder of the central nervous system (CNS), characterized by multiple areas of demyelination, especially in the brain, optic nerve, and spinal cord. It is one of the leading causes of disability in the young population, affecting more than 2.8 million people worldwide [1]. The underlying pathophysiology of MS is multifactorial and is thought to arise from complex interactions between genetic susceptibility, immune dysfunction, and environmental factors [2].

Interleukins (ILs) are a part of a diverse group of cytokines secreted primarily by innate and adaptive immune cells such as leukocytes, T lymphocytes, and macrophages. These act through specific cell surface receptors to control immune cell signaling pathways [3]. As MS is generally believed to arise from immune system dysfunction, accompanied by inflammatory processes, an imbalance in cytokine profiles, particularly increased levels of pro-inflammatory cytokines and decreased levels of anti-inflammatory cytokines, is thought to be involved in disease pathogenesis. It has been shown that elevated serum levels of several interleukins and cytokine receptors have been observed in patients with MS [4]. Cumulative evidence has demonstrated that IL18 and tumor necrosis factor-alpha (TNFα), proinflammatory cytokines, are significantly increased in the sera of MS patients during the remission and progressive phases of disease [5,6]. The elevation of IL18 enhances Th1 differentiation and induces the secretion of interferon-γ and TNFα, thereby enhancing demyelinating inflammation [5]. Similarly, the higher circulatory levels of TNFα disrupt the blood–brain barrier, causing oligodendrocyte apoptosis and axonal degeneration leading to irreversible neurological disability [6].

Circulating soluble cytokine receptors also contribute to the pathogenesis of MS, as they regulate not only the bioavailability of their corresponding cytokines but also the balance between autoimmunity and immunotolerance. It has been shown that soluble IL2 receptor alpha (sIL2Rα) levels are markedly elevated in serum, showing increased T-cell activation and impaired regulatory control. Studies have shown that higher sIL2Rα concentrations correlate with Expanded Disability Status Scale (EDSS) scores and MRI lesion load, providing evidence of disease activity [7]. Similarly, soluble IL7 receptor alpha (sIL7Rα), involved in lymphocyte survival and homeostasis, is upregulated in MS, and increased serum level of sIL7Rα leads to enhanced T-cell signaling and impaired peripheral tolerance [8]. Insulin-like growth factor-1 (IGF-1), a neurotrophic peptide, plays an important role in neuronal growth, survival, oligodendrocyte differentiation, and myelin repair [9]. Numerous studies have revealed significantly decreased serum IGF-1 levels in MS patients compared to healthy controls, suggesting that impaired IGF-1 signaling may contribute to demyelination and neurodegeneration [10].

Given the importance of these cytokines and soluble receptors, it seems appropriate to assess their serum levels as a potential tool not only for diagnosis, but also for evaluating the disease progression and severity. Moreover, elucidating the specific roles of cytokines and receptors in MS may facilitate the identification of potential novel therapeutic targets [11,12]. Therefore, we selected a panel of pro-inflammatory cytokines (IL18 and TNFα), cytokine receptors (sIL2Rα and sIL7Rα), and IGF-1 as potential serum biomarkers for the diagnosis of MS as a primary end-point in the Pakistani population. As secondary end-points, we also evaluated whether phenotypic patterns or severity of disease had any impact on serum levels of these cytokines.

## 2. Materials and Methods

Ethical Consideration: The study was a single-center, case–control study conducted at the Department of Neurology and department of Biomedical Sciences, King Edward Medical University (KEMU)/Mayo Hospital (a tertiary care hospital), Lahore, Pakistan. The duration of the study was from January 2024 to March 2025. The study was conducted as per the Declaration of Helsinki and after approval from the Institutional Review Board, KEMU, Lahore. After explaining, written consent was taken from all participants before the start of the study.

**Figure 1:**
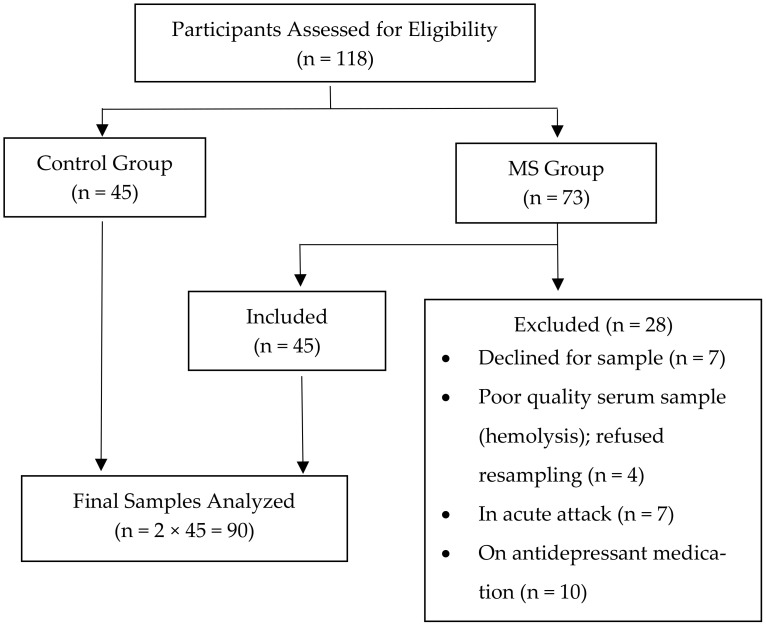
Flowchart of participants.

Patient inclusion, exclusion Criteria: The study was conducted on 45 MS patients (age 18 to 55 years), regardless of gender, after fulfilling the revised McDonald Criteria of 2017 [13]. The clinical and neurological examinations were carried out from 10.00 AM to 11.00 AM. We also recruited 45 age and sex-matched healthy individuals from the same hospital (Figure 1). The sample size was calculated using the following formula:

n = σ^2^(z_1−α_ + z_1−β_)^2^/(μ_1_ − μ_2_)^2^

whereas σ^2^ stands for variance. The values for confidence level (z_1−α_), power of test, 95% (z_1−β_), population mean 1 (µ_1_), and population mean 2 (µ_2_) were 1.96, 95%, 160, and 192, respectively.

The exclusion criteria were patients under the age of 18 or with an uncertain neurological diagnosis. Patients with recent MS relapse, corticosteroid use within 3 months, MS mimics (e.g., neuromyelitis optica, acute disseminated encephalomyelitis, tuberculous meningitis, vasculitis), radiologically/clinically isolated syndrome, other chronic inflammatory neurological disorders, concomitant diseases (e.g., IBD, gynecological/celiac/endocrine disorders, neoplasm, depression, hepatic/renal/cardiac issues, treatment with cytostatic drugs at any time), or pregnancy/lactation were excluded from the study.

After obtaining demographic data from patients and the healthy group, clinical data from MS patients were collected, including gender, clinical features, disease duration, disease pattern, number of relapses, age at illness onset, history of disease-modifying therapy (DMT), and clinical manifestations. The severity of the MS was also determined as a disease disability using the Extended Disability Status Scale (EDSS) as described earlier [14]. The EDSS score, which ranges from 0 (normal neurological examination) to 10 (death from MS) with 0.5-point increments, is derived from a detailed neurological examination and assessment of functional impairment. The severity of disease, based on EDSS score, was categorized as mild (0–3.5), moderate (4.5–6.5), and severe (≥7.0). The phenotype pattern of the disease was also determined as per the definition [15]. Clinically Isolated Syndrome (CIS) is defined as the presence of a first clinical attack of MS with objective evidence of neurological changes and a lesion compatible with MRI studies. The relapse remittance (RR) pattern means a history of at least two clinical attacks or at least one clinical attack with new documented MRI lesions over time. Relapse was defined as an acute deterioration of neurological function lasting for at least 24 h followed by a period of total or partial recovery. The secondary progressive (SP) is evolving in patients previously with a history of clinical attack (RRMS) who demonstrated gradual and progressive disability with or without periods of relapse. The primary progressive (PP) is a gradual and progressive worsening of neurological function from the beginning of the disease, with or without subsequent relapses observed for at least one year.

Measurement of cytokines in serum samples: The venous blood samples (5.0 mL) were collected in the morning after an overnight fasting of about 8–12 h to avoid circadian variations. The blood samples were centrifuged at 3000 rpm for 15 min to recover the serum. The obtained serum samples were stored at −80 °C until analyzed. The concentrations of cytokines in the serum samples were measured using enzyme-linked immunosorbent assay (ELISA)-based commercial kits (Invitrogen™, Thermo Fisher Scientific Inc., Waltham, MA, USA) according to the manufacturer’s instructions. The absorption, which is proportional to the cytokine concentration, was measured calorimetrically using an ELISA plate reader (Thermo Scientific Multiskan EX, Thermo Fisher Scientific Inc., Waltham, MA, USA) at an absorbance of 450 nm. The cytokines/cytokine receptor alpha (Rα) subunits measured were IGF-1, IL18, sIL2Rα, sIL7Rα, and TNFα. As per the manufacturer’s information provided, the minimum detectable concentrations, also called assay sensitivity, assay range, interassay CV, and intraassay CV of IGF-1 (Catalog No. EH250RB) were 0.1 ng/mL, 0.1–30 ng/mL, <12%, and <10%, respectively. For IL18 (Catalog No. BMS267-2), the values of assay sensitivity, assay range, interassay CV, and intraassay CV were 9.0 pg/mL, 78–5000 pg/mL, 8.1%, and 6.5%, respectively. The values for assay sensitivity, assay range, interassay CV, and intraassay CV of sIL7Rα (Catalog No. EH276RB) as reported by the manufacturer were 0.14 ng/mL, 0.14–100 ng/mL, <12%, and <10%, respectively. For TNFα (Catalog No. BMS2034), assay sensitivity, assay range, interassay CV, and intraassay CV were 5.0 pg/mL, 23–1500, 8.1%, and 7.7%, respectively. For sIL2Rα (Catalog No. ECIL2RA), values of 15.0 pg/mL, 15–400 pg/mL, <12%, and <10% were for analytical assay sensitivity, assay range, interassay CV, and intraassay CV, respectively. Samples with values outside the detection range were appropriately diluted and re-assayed. All the samples were run in duplicate, and none of the tested samples were below the limit of detection. Serum could not be separated due to hemolysis in 4 samples, and participants were not included in the study (Figure 1). The quantitative analysis of cytokines was carried out by a person who was blinded to the sample identity to minimize the potential bias.

Statistical Analysis: Data were analyzed on the Statistical Package for Social Sciences (SPSS version 20.4, IBM Corp., Armonk, NY, USA). The normality of the data was determined using the Kolmogorov–Smirnov test. Categorical data are expressed as frequencies and percentages. The continuous variables are expressed as mean and standard deviation (mean ± SD) or median (IQR), whichever is appropriate. Chi-square test was used to compare the association of categorical data. An independent *t*-test or Mann–Whitney U test was used to compare the means between two groups based on the normality of the data. To compare various phenotypic patterns of the disease, one-way ANOVA was used to compare the means between groups with Tukey’s test as a post hoc test for intergroup comparison if the data were normally distributed. Otherwise, the Kruskal–Wallis test was used for non-normally distributed data. Dunn’s test, as a post hoc test with Bonferroni correction, was used for pairwise comparison of means. Pearson’s correlations or Spearman’s correlations analysis, whichever is appropriate, was used to determine the association between cytokines and EDSS, age, and disease duration. To evaluate the association of serum cytokine levels with MS status (MS vs. healthy), each cytokine was analyzed separately using a multivariable binary logistic regression. Two series of multivariate linear regression models were constructed using the “Enter method,” in which all the independent variables were entered into the model simultaneously to predict the outcome. In the first series, each cytokine was treated as the dependent variable, and group (both MS and healthy control) and DMT were treated as confounders. DMT was coded as 0 (no treatment) and 1 (treatment) for the MS group. Moreover, healthy control subjects were also coded 0 for all subjects. Because the study followed a case–control design and the groups were matched for age and sex, these variables were not included in the model. Moreover, disease phenotype was not included as an independent variable in the final model due to the very low number of subjects within the respective sub-groups. Multicollinearity was assessed using variance inflation factors (VIF). The second series of a multivariate linear regression model was constructed exclusively for the MS group. Again, each cytokine served as a dependent variable. The EDSS, disease duration, use of DMT, and age when first symptoms appeared were included as independent variables. Age was initially considered but was excluded from the final models due to strong multicollinearity with the studied predictors, as indicated by elevated VIF (>50). The predictive power of the serum biomarkers was calculated by plotting receiver-operating characteristic (ROC) curves (a graphical presentation of sensitivity versus 1-specificity) and also defining the area under the curve (AUC). The AUC is regarded as an indicator for evaluating the accuracy of a diagnostic test. The AUC value varies from 0 to 1, being close to 1 when the diagnostic assay has a high accuracy. The optimal cut-off values for each cytokine were calculated using the Youden Index (J) for each threshold using J = Sensitivity − (1 − Specificity). The significance level was predetermined at *p* < 0.05.

## 3. Results

The mean age (years) for MS patients (33.24 ± 9.84; range 18–55) and healthy controls (33.24 ± 9.79; Range 18–55) were comparable (*p* > 0.05). Similarly, the number of males was 19 (42.22%), and the number of females was 26 (57.77%) in both the MS and healthy control groups. The clinical spectrum of MS patients is summarized in Table 1. Twenty-one patients presented with a single clinical symptom of MS, whereas 24 patients had multiple clinical symptoms and signs.

Of 45 MS patients, 25 (55.55%) were <35 years old, and 20 (44.44%) were >35 years old. Additionally, 29 (64.44%) MS patients were on DMT, primarily ocrelizumab (93.33%) and azathioprine (6.66%). Table 2 represents the patient characteristics across the three phenotypic patterns observed in the MS group. Most patients were in the relapsing–remitting (RR) group (n = 34) than in the secondary progressive (SP; n = 5) or primary progressive (PP; n = 6) groups. The MS patients were younger (*p* < 0.05) in the PP group than in the RR group. The EDSS score was higher (*p* < 0.05) in the SP than in the RR patients. Based on the EDSS score, the disease was mild in 24 (53.33%) patients, moderate in 19 (42.22%), and severe in only 2.0 (4.44%) patients.

As the disease was more prevalent in females, the data were further stratified based on gender. As shown in Table 3, there were no differences in age, age when symptoms appeared for the first time, and EDSS between males and females in MS patients. However, the duration of disease was more prolonged (*p* = 0.006) in male than in female MS patients.

**Figure 2:**
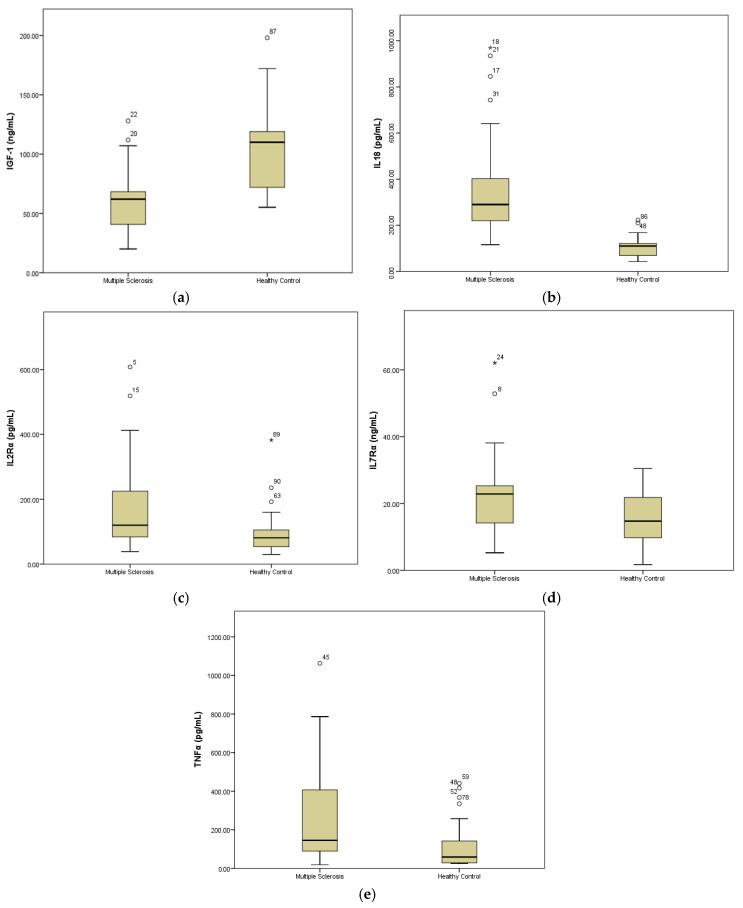
The serum concentrations of IGF (**a**), IL18 (**b**), sIL2Rα (**c**), sIL7Rα (**d**), and TNFα (**e**) in MS (n = 45) and healthy control (n = 45) groups. Statistics for IGF-1 (*p* < 0.001), IL18 (*p* < 0.001), sIL2Rα (*p* < 0.001), and sIL7Rα (*p* < 0.01) are computed with an independent *t*-test. For TNFα, the Mann–Whitney test was used to determine the *p*-value (*p* < 0.01) between the MS and healthy control groups. n = 45/group. * shows the extreme outliers.

The comparison of TNFα, IL18, sIL2Rα, sIL7Rα, and IGF-1 between the MS and healthy control groups is illustrated in Figure 2. The serum levels of pro-inflammatory cytokines (IL18 and TNFα) were significantly higher (*p* < 0.001; *p* < 0.01) in the MS group compared to the healthy control group. Similarly, sIL2Rα and sIL7Rα were also higher (*p* < 0.01) in the MS group than in the healthy control individuals. On the other hand, serum level of IGF-1 was higher (*p* < 0.001) in the healthy individuals when compared with the MS group.

When serum levels of cytokines and cytokine receptor subunits were assessed based on the phenotypic pattern of disease, no significant differences were noted except for lower (*p* < 0.01) IGF-1 in the PP group compared to the other groups (Table 4).

The serum levels of selected biomarkers based on the severity of the disease, as determined by EDSS, are mentioned in Table 5. The severity of the disease did not affect the serum profile of cytokines or cytokine receptor subunits except for IGF-1, which was significantly lower (*p* < 0.004) in the moderate type of disease compared to the mild form. The circulating levels of cytokines were not influenced by the DMT. The mean serum concentrations in patients receiving DMT versus those without treatment were comparable (*p* > 0.05) for IGF-1 (65.55 ± 25.68 vs. 55.36 ± 14.83 (ng/mL), IL18 (348.28 ± 215.74 vs. 355.69 ± 214.74 pg/mL), sIL2Rα (190.92 ± 150.71 vs. 160.50 ± 103.27 pg/mL), sIL7α (22.49 ± 12.98 vs. 19.91 ± 6.22 ng/mL) and TNFα (253.84 ± 264.93 vs. 252.32 ± 191.28 pg/mL).

As shown in Table 6, DMT was not a significant confounder in any of the regression models when analyzed separately for each cytokine as the dependent variable. In contrast, all cytokines except sIL7Rα exhibited significant differences from the healthy control group, which served as a reference for regression models. We did not include age and gender as confounders in all models, as the study was an age and gender-matched case–control study.

Results of models constructed for each cytokine as a dependent variable for the MS group with EDSS, duration of disease, age when first-time symptoms appeared, and use of DMT as confounders are shown in Table 7. None of the confounders influenced the levels of circulating cytokines in the MS patients except sIL2Rα. However, longer duration of disease was independently associated with lower IL2Rα levels (B = −8.13, 95% CI −16.07 to −0.19, *p* < 0.05), after adjustment for EDSS, DMT, and age at symptom onset.

**Figure 3:**
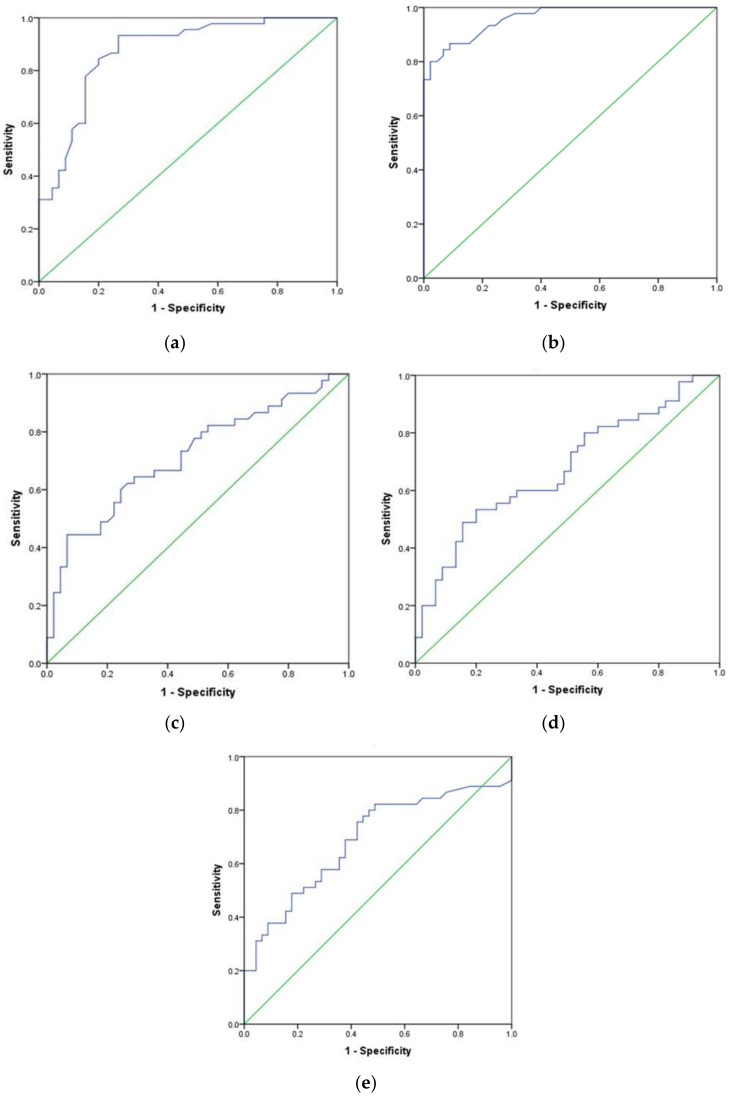
Receiver operating characteristic (ROC) curves constructed for IGF-1 (**a**), IL18 (**b**), sIL2Rα (**c**), sIL7Rα (**d**), and TNFα (**e**). Statistics for IGF-1 (*p* < 0.001), IL18 (*p* < 0.001), sIL2Rα (*p* < 0.001), and sIL7Rα (*p* < 0.01). n = 45/group.

We also constructed ROC curves to discriminate the potential use of various cytokines and cytokine receptor subunits for the diagnosis or prognosis of MS (Figure 3). IL18 showed excellent (*p* < 0.001) discriminator power for the diagnosis of MS (cut-off ≥ 165.50 pg/mL; sensitivity 84.4%, specificity 93.3%; Youden index = 0.777) as the AUC was 0.96 ± 0.017 (95% CI = 0.927–0.994), followed by IGF-1 (*p* < 0.001) with an AUC of 0.873 ± 0.037 (95% CI = 0.799–0.946; cut-off ≤ 68.70 ng/mL; sensitivity 77.8%, specificity 84.4%; Youden index = 0.622). The sIL2Rα has fair (*p* < 0.001) diagnostic power (AUC = 0.717 ± 0.054; 95% CI = 0.611–0.823) with its cut-off value of 98.90 pg/mL, yielding a sensitivity of 64.4% and specificity of 71.1% (Youden index = 0.355). Similarly, TNFα also showed moderate discriminatory (*p* < 0.001; AUC = 0.687 ± 0.056; 95% CI = 0.575–0.799) ability with a cut-off value of ≥67.80 pg/mL, and the sensitivity, specificity, and Youden index were 82.2%, 51.1%, and 0.333, respectively. On the other hand, ROC curve analysis identified an optimal sIL7Rα cut-off value of ≥24.89 ng/mL, yielding a sensitivity of 33.3% and specificity of 91.1% (Youden index = 0.244), indicating limited discriminatory (*p* < 0.01) ability (AUC = 0.675 ± 0.057; 95% CI = 0.564–0.786).

### Correlation Analysis

The correlation coefficients of various variables are presented in Table 8. A moderate positive correlation (*p* < 0.01) was observed between EDSS and duration of disease. Interestingly, we observed a significantly (*p* = 0.004) moderate negative correlation (r = −0.418) between IGF-1 and EDSS. A strong (*p* < 0.001; r = 0.81) correlation was noted between age and age at the first onset of MS. 

## 4. Discussion

In this study, we investigated the serum levels of a panel of pro-inflammatory cytokines, cytokine receptors, and IGF-1 in MS patients as a diagnostic tool, along with their utility to differentiate various stages of the disease. IL18, also known as interferon-γ-inducing factor, is a pro-inflammatory cytokine primarily produced by monocytes and macrophages. The normal functioning of the CNS requires low levels of IL18, as its deficiency can lead to CNS dysfunction, but its overwhelming release for a longer period of time may be toxic to the CNS [16]. Our study revealed higher serum levels of IL18 in MS patients compared to healthy individuals. Similar to our results, a body of evidence demonstrated higher circulating levels of IL18 in MS patients [17,18,19,20]. On the contrary, others reported no difference in IL18 serum levels between RRMS patients with acute exacerbation and healthy controls [21]. The rise in IL18 is also associated with an active form of the disease, as it has been demonstrated that IL18 is more elevated in MS patients with active MRI lesions than those MS patients without lesions using poser’s criteria [17]. It appears that this overwhelming secretion of IL18 might have caused damage in the CNS of MS patients, suggesting an active involvement in the pathogenesis of the disease. On the contrary, several studies have reported no increase in serum IL18 levels in patients with MS compared with healthy controls. For example, Orhan et al. (2016) found no significant difference in IL18 concentrations between MS patients and control individuals in the Turkish population [22]. Similarly, Trenova et al. (2018) reported no difference in serum IL18 levels between patients with relapsing–remitting MS and controls in a Bulgarian population [23]. In the current study, no differences in serum IL18 were observed among MS phenotypes, in contrast to Nicoletti et al. (2001), who reported higher circulating IL18 levels in secondary progressive MS than in RRMS using Poser’s criteria for phenotype classification [18]. Our study could not find any significant correlations of IL18 with age, sex, EDSS, or duration of disease. These results are consistent with other studies that also found no significant correlations between IL18 levels and age, gender, EDSS, disease duration, or age at first onset of MS [18,19]. The lack of association between circulatory IL18 levels and these clinical markers remains unclear, and the underlying mechanisms of this observation have yet to be elucidated. Based on the previous studies of elevated circulating IL18 levels in MS patients, we investigated the utility of IL18 as a diagnostic biomarker for the diagnosis of MS. Our results indicate that IL18 may be a promising biomarker for MS, as it showed a strong diagnostic performance with excellent discriminative power (*p* < 0.001), reflected by a high AUC when analyzed for the ROC curve. Others have also suggested that a higher serum level of circulating IL18 in MS patients with active MRI lesions compared to non-active MS lesions makes IL18 a suitable biomarker for the diagnosis and monitoring of disease progression [17,24].

This study observed elevated TNFα levels in MS patients’ sera compared to healthy controls. These findings are consistent with previous reports of higher serum TNF-α in MS patients versus healthy individuals [25,26,27]. Similarly, Alves-Leon et al. (2001) also reported higher TNFα in the serum samples of MS patients compared to healthy individual independent of the stage of disease [28]. In another study, there was increased production of TNFα from the blood of MS patients preceding disease activity [29]. In this study, serum levels of TNFα were numerically higher in PPMS (347.51 ± 374.71) than in RRMS (236.48 ± 213.01) or SPMS (254.63 ± 250.97), but statistical significance was not established due to high SD. Existing evidence shows no significant differences in serum levels of TNFα between RRMS patients and healthy controls [4,30]. Findings regarding serum TNFα levels across different stages of clinical disability have been inconsistent. In contrast to our results, lower serum TNFα levels in MS patients have been demonstrated in remission compared with those in the relapse course of disease [24,30,31]. The apparent differences regarding the serum levels of TNFα in various studies might be due to the criteria for diagnosis, severity of disease, types of DMTs, technique of analysis, types of ELISA kit used, race of patients, and also the genotypes of patients. A MS Genome-Wide Association Study identified a single nucleotide variant in the *TNFRSF1A* gene (encoding TNFR1) that has been associated with increased risk of MS development through enhancing expression of sTNFR1 and increasing serum levels of TNFα [32]. Higher levels of serum TNFα, as observed in our studies, have more affinity for TNFR1 receptors, resulting in microglial activation, oligodendrocyte disruption, and demyelination. Combined with the potential role of the TNF/TNFR1 signaling pathway in the immunogenicity of MS, elevated circulating TNFα could potentially serve as a diagnostic biomarker or progression indicator. However, as a cross-sectional study, we could only assess its diagnostic utility, which showed poor discriminatory power (AUC = 0.687). In consistent to our results, Vladić Anton (2002) found that soluble proteins of TNFα, as assayed by monoclonal antibody-based ELISA, could not serve as markers of MS activity [33]. On the other hand, another study found that TNFα is a good predictor of MS diagnosis correctly classifying approximately 97% subjects with 100% sensitivity and 79% specificity [27], but was insufficient to distinguish the clinical form of MS. Because of inconsistent results, a longitudinal study with a larger cohort and careful control of confounding variables is warranted to further evaluate the potential of TNFα as a diagnostic and prognostic biomarker in MS.

Presently, elevated serum levels of sIL2Rα have been found in MS patients compared to control individuals. Consistent with our findings, a body of evidence demonstrated the higher circulating levels of sIL2Rα in MS patients compared to the control group [34,35,36,37,38]. In our study, we were unable to find any differences in the serum sIL2Rα levels in various subtypes of MS. Similar to our findings, others also found no significant differences in the serum levels of sIL2Rα in RR, SP, and PP subtypes of MS [38,39]. Trotter et al. (1991) could not find any significant differences in serum levels of sIL2Rα in MS patients with chronic progressive subtypes compared to healthy control subjects [40]. Similarly, another study reported elevated serum sIL2Rα levels in relapsing MS patients but not in remission, compared to controls, suggesting sIL2Rα levels may reflect disease activity or severity [36,37]. The higher level of sIL2Rα can bind with IL2, thereby affecting suppressor cell functions that are involved in the pathogenicity of MS. The sIL2Rα/IL2 interaction might have reduced the functional activity of Treg cells that play a pivotal role in MS. This assumption has been supported by another study where higher circulating sIL2Rα has been found to enhance the proliferation and expansion of CD41 T cells that are involved in the demyelination and neurodegenerative changes [38].

The circulating sIL2Rα is generated by shedding of the IL2Rα chain from the surface of activated T cells that can combine with IL2 in the circulation. The sIL2Rα/IL2 interaction initiates signaling pathways that are crucial in maintaining balance between immune activation and immunotolerance. In MS, this balance is disrupted, destroying myelin proteins. Recent studies have shown that elevated sIL2Rα has been implicated not only in aggravating MS but also acts as an important risk factor for developing MS [41,42,43]. Sharief and Thompson (1993) suggested that measurement of sIL2α concentrations may provide an objective marker of disease activity in patients with MS [44]. Given its role as a risk factor and potential therapeutic target (via IL2Rα antagonism), we examined sIL2Rα as a diagnostic biomarker for MS. ROC analysis showed fair discriminatory performance for distinguishing MS patients from controls (AUC ≈ 0.71). Because of its modest AUC value and the lack of significant differences in serum levels across various MS phenotypes, the utility of circulating sIL2Rα as a prognostic biomarker or a tool for monitoring treatment response remains limited. Moreover, as this study was cross-sectional in design, longitudinal investigations with larger sample sizes are warranted to determine its potential role as a diagnostic and prognostic biomarker, as well as in assessing therapeutic efficacy in MS.

The IL7α receptors for IL7 are present on the surface of B and T lymphocytes. The IL7/IL7Rα interaction plays a pivotal role in the survival and development of T cells and homeostasis of T and B cells, particularly CD4^+^T cells [45]. In our study, we demonstrated higher serum levels of sIL7Rα in MS patients compared to healthy individuals without any significant changes in the various phenotypes of MS. Data regarding the plasma levels of sIL7Rα in MS are scarce. In contrast to our findings, a lower level of plasma-free sIL7Rα, with a higher ratio of free to membrane-bounded IL7Rα, has been reported in the MS patients compared to healthy subjects [8]. However, considerable variability in IL2Rα expression in lymphocytes of patients with MS has been reported in the literature. Bina et al. (2017) could not find any difference in mRNA expression between healthy and MS patients for both soluble and membrane-bound IL7Rα in the Iranian population [46]. On the other hand, increased expression of IL7Rα in PBMCs of RRMS patients has also been reported in the USA cohort [47]. These differences in IL7Rα expression may be associated with specific genotypes, since the C allele has been identified as a stronger risk factor for MS than the T allele [48]. It has been reported that different IL7Rα genotypes in both control subjects and MS patients are associated with variable plasma concentrations of sIL7Rα, with higher levels in the CC genotype compared with the TT genotype [49]. However, the study did not compare the sIL7Rα concentrations between MS patients and controls across genotypes. Elevated levels of sIL7Rα can bind circulating IL7, increasing its overall bioavailability in plasma and changing the sIL7Rα/IL7 signaling pathway. While this interaction may initially reduce the amount of IL7 available for membrane-bound IL7RαA, the sIL7Rα/IL7 complex can act as a reservoir that may continuously release IL7. This sustained signaling might have shifted lymphocyte responses from immune tolerance toward a proinflammatory, autoimmune state, ultimately contributing to demyelination within the CNS. An in vitro study supports our hypothesis, showing that sIL7Rα reduced IL7 consumption in IL7-dependent E28 cells, increasing IL7 bioavailability and suggesting agonistic effects on IL7 signaling [50]. Consistent with our results, no significant associations were observed between sIL7Rα and disease duration, EDSS, or age at disease onset in MS patients [51]. Since the levels of soluble cytokine receptor levels are altered in many autoimmune diseases [48], we examined whether serum sIL2Rα could be used as a diagnostic biomarker in MS. Our findings were not encouraging, as the discriminative value (AUC = 0.675) of this biomarker was low when analyzed by ROC curve. Imani et al. (2021) also evaluated IL7Rα expression as a diagnostic biomarker for MS and reported its limited diagnostic utility, with an AUC of 0.66, which closely aligns with our findings [52].

IGF-1 has been implicated in the myelinogenic activity and development of oligodendrocytes, thereby helping in the survival of both oligodendrocytes and neurons in the central nervous system [53]. Our study identified lower serum IGF-1 levels in the MS group than in healthy individuals, which were higher in RRMS and SPMM patients compared to PPMS patients. There is some variation in the literature regarding the serum levels of IGF-1 in MS. For example, the mRNA expression of IGF-1 was higher in healthy individuals than in MS patients in an Iranian cohort [54]. On the other hand, several studies failed to demonstrate differences in IGF-1 levels between MS and healthy patients [53,55,56,57,58]. A few studies reported higher serum IGF-1 levels in MS patients than in control subjects [59,60,61]. These differences in the serum IGF-1 levels may be due to several factors, such as age, EDSS, duration of disease, subtype of disease, type of treatment, and particular genotype, as MS patients with genotype C/C significantly produced less IGF-1 than those with genotype T/T [54]. For instance, a lower serum IGF-1 was detected in MS patients older than 50 years compared with MS patients <50 years [62]. MS patients receiving DMT showed higher IGF-1 levels compared to MS patients without any medication [59]. However, our study did not reveal any role of DMT in circulatory cytokines or cytokine receptors in MS patients. Another study described low circulatory IGF-1 levels in patients with primary progressive MS and those with high EDSS ≥ 6 and disease duration > 5 years [63]. The lower level of IGF-1 reported in the present study might be due to a higher level of IGF-binding protein that binds with circulating IGF-1, leading to low serum availability, as IGF-1 binding proteins are found to be elevated in MS, causing lower bioavailability of IGF [57]. The lower IGF-1 might be involved in the pathogenesis of MS, as transgenic mice that overexpressed IGF-1 had increased myelin content in their CNS, while IGF-deficient mice or a functional mutation in the IGF-1 gene showed a marked reduction in both myelination and the numbers of oligodendrocytes [64,65,66,67]. We observed higher IGF-1 levels in patients with mild MS disease compared to moderate disease severity, along with a moderate inverse correlation between IGF-1 and EDSS. These findings are in line with those of Akçeli and co-workers, who demonstrated negative associations between IGF-1 and age, EDSS, relapse frequency, and disease duration [58]. Our findings indicate that IGF-1 has good diagnostic performance for MS, as demonstrated by an AUC exceeding 0.80. On the other hand, Nageeb and co-workers (2018) suggested that serum IGF-I levels should not be considered diagnostic biomarkers of disease, but rather prognostic biomarkers [63]. Nevertheless, the utility of IGF-1 in distinguishing MS from healthy individuals remains unclear and warrants further investigation.

## 5. Conclusions

In conclusion, we demonstrated that higher circulatory levels of IL18, TNF, IL2Rα, and IL7Rα were noted in MS patients. The protective IGF-1 concentration was lower in MS patients. ROC analysis showed that IL18 and IGF-1 were excellent diagnostic biomarkers, followed by sIL2Rα, which has fair discriminatory diagnostic power. Contrary to our expectations, sIL7Rα and TNFα did not prove to be useful diagnostic tools for discriminating MS. From the above, it appears that circulatory ILs and soluble cytokine receptors act as myelinotoxic factors by triggering immune-inflammatory processes. Although lower IGF-1 levels were observed in PPMS (n = 6) compared with other forms, no definitive inference can be made due to the very small sample size. DMT, disease duration, and EDSS did not influence circulating biomarker levels, which may be related to the study design, as this was a single-time-point observational study. Therefore, the results of the present study should be interpreted in light of several limitations. As this study is observational in nature, one should be very cautious in drawing causal inferences. Another limitation is the low sample size, especially since only five and six patients were included in the SPMS and PPMS groups, respectively. Interpretation of severity-related findings should be done cautiously, since the EDSS was used as the sole measure of disability and has known limitations, focusing more on walking. Therefore, longitudinal studies with a larger cohort may be conducted to assess the utility of these and other possible cytokines and their soluble receptors as both diagnostic and prognostic biomarkers for MS. Moreover, it will be more informative to measure not only the circulatory levels of cytokines but also their corresponding soluble and membrane-bound receptors for a better understanding of signaling pathways, and also for a deeper insight into the pathogenicity of MS. Future studies using larger, longitudinal cohorts and multi-marker panels are warranted to validate these findings and to determine whether integrating cytokine profiles with imaging or genetic markers can enhance prognostic accuracy.

## Figures and Tables

**Table 1:** Clinical spectrum of MS patients (n = 45).

Clinical Characteristics	Frequency	Percentage	Statistics
Single Clinical Sign (yes); n = 21			*p* = 0.533
Myelitis (My)	7	33.33	d_f_ = 6
Recurrent myelitis	1	4.76	π^2^ = 12.44
Optic nerve impairment (ON)	5	23.80	
Recurrent optic nerve	3	14.28	
INO	1	4.76	
Hemiplegia (HP)	3	14.28	
Monoplegia (MP)	1	4.76	
Double Clinical signs (yes); n = 24			*p* = 0.001
Myelitis + ON	11	45.83	d_f_ = 5
Myelitis + INO	1	4.16	π^2^ = 21.37
ON + Fascial Palsy	1	4.16	
ON + HP	5	20.83	
ON + Ataxia	3	12.50	
HP + Ataxia	3	12.50	

INO = Internuclear ophthalmoplegia; MS = Multiple sclerosis.

**Table 2:** Characteristics of MS patients based on disease course/phenotypic pattern.

Parameter	^†^ MS (n = 45)	Disease Course: Mean ± SD (95% CI)			Statistics
		Relapsing-Remitting (n = 34)	Secondary Progressive (n = 5)	Primary Progressive (n = 6)	
Gender; n (%) Male Female	19 (42.22) 26 (57.77)	15 (44.11) 19 (55.88)	2 (40.00) 3 (60.00)	2 (33.33) 4 (66.66)	*p* = 0.881 χ^2^ = 0.255
DMT, n (%) Yes No	29 (64.44) 16 (35.55)	20 (58.83) 14 (41.17)	5 (100.00) 0 (0.00)	4 (66.66) 2 (33.33)	*p* = 0.198 χ^2^ = 3.240
Age (years)	33.24 ± 9.84 (30.28–36.20)	34.44 ± 9.50 ^a^ (30.12–36.75)	41.40 ± 9.34 ^ab^ (29.79–53.00)	25.33 ±6.74 ^b^ (18.25–32.40)	*p* = 0.022
Duration of MS (years)	7.48 ± 5.80 (5.74–9.23)	7.11 ± 5.76 (5.10–9.12)	12.00 ± 6.04 (4.49–19.50)	5.83 ± 4.87 (0.71–10.94)	*p* = 0.163
Age at first symptom (years)	25.82 ± 7.54 (23.55–28.08	26.35 ± 7.67 (5.10–9.12)	12.00 ± 6.04 (20.46–38.33)	5.83 ± 4.87 (16.05–23.61)	*p* = 0.076
EDSS	3.57 ± 2.00 (2.97–4.18)	3.08 ± 1.97 ^b^ (2.39–3.77)	5.30 ± 1.97 ^a^ (2.39–3.77)	4.91 ± 1.15 ^ab^ (3.70–6.12)	*p* = 0.012

^†^ *p*-values are calculated only among sub-types based on phenotypic pattern, excluding the multiple sclerosis (MS) group. DMT = Disease-modifying treatment. EDSS = Expanded disability status scale. ^a,b^ Means sharing the different superscripts differ significantly at *p* < 0.05. Post hoc was the Duncan multiple range test.

**Table 3:** Distribution of age, duration of disease, and EDSS in patients based on gender.

Parameter	(Mean ± SD)		*p*-Value
	Male (n = 19)	Female (n = 26)	
Age (years)	35.89 ± 9.06	31.30 ± 10.11	0.124
Duration of disease (years)	10.26 ± 6.23	5.58 ± 4.60	0.006
Age at first onset (years)	25.84 ± 6.90	26.00 ± 7.87	0.945
EDSS	4.28 ± 1.71	4.00 ± 1.58	0.563

EDSS = Expanded disability status score.

**Table 4:** Serum concentrations of biomarkers based on phenotypic pattern of MS.

Marker	Mean ± SD (95% CI)			^†^ *p*-Value
	Relapsing–Remitting (n = 34)	Secondary Progressive (n = 5)	Primary Progressive (n = 6)	
TNFα (pg/mL)	236.48 ± 213.01 (162.16–310.81)	254.63 ± 250.97 (−56.99–566.25)	347.51 ± 374.71 (−45.73–740.75)	0.588
IL18 (pg/mL)	359.48 ± 238.38 (276.30–442.66)	349.04 ± 136.12 (180.01–518.06)	303.98 ± 77.99 (222.13–385.83)	0.847
sIL7Rα (ng/mL)	22.45 ± 11.76 (18.34–26.55)	16.02 ± 8.69 (5.23–26.82)	21.27 ± 8.05 (12.82–29.72)	0.488
sIL2Rα (pg/mL)	166.31 ± 131.81 (120.32–212.30)	213.75 ± 147.67 (30.38–397.12)	230.24 ± 153.42 (69.23–391.25)	0.486
IGF-1 (ng/mL)	63.34 ± 18.42 ^a^ (57.91–70.77)	44.20 ± 35.39 ^a^ (0.25–88.14)	38.91 ± 16.45 ^b^ (21.65–56.18)	0.004 ^†^

MS = Multiple sclerosis; IGF-1 = Insulin-like growth factor, IL = Interleukin; TNFα = Tumor necrosis factor-alpha; sILR= Soluble interleukin receptor; CI = Confidence interval. *p*-values for each variable were calculated using 1-way ANOVA with Tukey’s test as a post hoc test for group comparisons. ^†^ For IGF-1, the Kruskal–Wallis test was used to calculate the *p*-value as the data were not normally distributed. Donn’s post hoc test with Bonferroni correction was used for pairwise comparisons. ^a,b^ Means sharing the different superscripts differ significantly at *p* < 0.05.

**Table 5:** Serum concentrations of various biomarkers based on severity of MS.

Marker	Mild (n = 24)	Moderate (n = 19)	^†^*p*-Value
TNFα (pg/mL)	283.25 ± 296.34	195.87 ± 137.05	0.242
IL18 (pg/mL)	330.65 ± 179.35	360.04 ± 217.29	0.629
sIL7Rα (ng/mL)	23.91 ± 13.16	18.92 ± 6.97	0.143
sIL2Rα (pg/mL)	181.02 ± 126.81	189.05 ± 153.07	0.852
^†^ IGF-1 (ng/mL)	64.55 ^a^ (59.55–71.42)	40.80 ^b^ (30.00–63.00)	0.003

The severity of Multiple Sclerosis (MS) based on EDSS score was categorized as mild (0–3.5), moderate (4.5–6.5), and severe (≥7.0). As the disease was severe only in 2 patients, the third group (severe group; n = 2) was not included in the statistical analysis due to underpower sample size. Values are expressed as mean ± SD. For IGF, which is expressed as Median (IQR). IGF-1 = Insulin-like growth factor; IL = Interleukin; sILR = Soluble interleukin receptor; TNFα = Tumor necrosis factor; EDSS = Expanded disability status scale. An independent *t*-test was used to calculate the *p*-value for all variables. ^†^ For IGF-1, the Mann–Whitney U test was used. ^ab^ Values not sharing the same superscripts differ significantly (*p* < 0.05).

**Table 6:** Summary of multivariate regression analysis models using each cytokine as a dependent variable.

Model	B	Std. Error	Beta	*p*-Value	95% CI for B (Limit)		Adjusted R^2^
					Lower	Upper	
IGF-1 MS DMT	50.283 10.197	8.489 9.083	0.694 0.132	0.000 0.265	33.410 −7.856	67.156 28.250	0.359 - -
IL18 MS DMT	−250.105 −7.404	44.838 47.973	−0.642 −0.018	0.000 0.878	−339.226 −102.756	−160.984 87.948	0.382 - -
sIL2Rα MS DMT	−67.440 30.423	30.629 32.770	−0.299 0.126	0.030 0.356	−128.318 −34.711	−6.562 95.557	0.138 - -
sIL7Rα MS DMT	−4.483 2.569	2.730 2.921	−0.230 0.123	0.104 0.382	−9.910 −3.237	0.943 8.374	0.086 - -
TNFα MS DMT	−144.653 1.527	54.369 58.171	−0.366 0.004	0.009 0.979	−252.718 −114.093	−36.587 117.147	0.116 - -

Healthy control (n = 45) served as the reference group for comparison with MS (n = 45). For DMT, subjects receiving no treatment served as a reference. IL = Interleukin, TNF = Tumor necrosis factor, CI = Confidence interval.

**Table 7:** Summary of multivariate regression analysis models using individual cytokines as dependent variables.

Model	B	Std. Error	Beta	*p*-Value	95% CI for B (Limit)		Adjusted R^2^
					Lower	Upper	
IGF-1 EDSS DMT Duration Onset-Age	−3.753 9.149 0.105 0.244	1.969 6.131 0.572 0.410	−0.316 0.220 0.030 0.088	0.064 0.143 0.856 0.554	−7.732 −3.242 −1.051 −0.583	0.226 21.540 1.260 1.072	0.067 - - - -
IL18 EDSS DMT Duration Onset-Age	9.590 −12.594 5.408 0.299	22.219 69.190 6.451 4.623	0.076 −0.029 0.147 0.010	0.668 0.856 0.407 0.949	−35.316 −152.432 −7.630 −9.043	54.496 127.245 18.446 9.642	0.058 - - - -
sIL2Rα EDSS DMT Duration Onset-Age	17.526 42.654 −8.128 1.322	13.530 42.132 3.928 2.815	0.219 0.153 −0.349 0.071	0.203 0.317 0.045 0.641	−9.819 −42.499 −16.067 −4.367	44.871 127.806 −0.188 7.011	0.028 - - - -
sIL7Rα EDSS DMT Duration Onset-Age	−1.513 2.663 −0.061 −0.344	1.090 3.395 0.317 0.227	−0.232 0.117 −0.032 −0.225	0.173 0.437 0.849 0.137	−3.716 −4.198 −0.700 −0.802	0.690 9.524 0.579 0.114	0.054 - - - -
TNFα EDSS DMT Duration Onset-Age	8.015 10.373 −1.503 −6.225	24.975 77.774 7.251 5.196	0.057 0.021 −0.037 −0.188	0.750 0.895 0.837 0.238	−42.462 −146.813 −16.159 −16.726	58.492 167.560 13.152 4.277	0.061 - - - -

For all confounders, each cytokine served as the dependent variable. IGF = Insulin-like growth factor; IL = Interleukin, sILR = soluble interleukin receptor; TNF = Tumor necrosis factor; CI = Confidence interval. EDSS = Expanded disability status scale. Onset-Age indicates the age at which symptoms first appeared. DMT = Disease-modifying therapy.

**Table 8:** Correlation coefficients of cytokines with various clinical variables (n = 45).

Variables	Correlation Coefficient (r)	*p*-Value
IGF-1 vs. Age	0.020	0.898
IGF-1 vs. Age at onset	0.155	0.310
IGF-1 vs. Duration	−0.134	0.381
IGF-1 vs. EDSS	−0.418	0.004
IL18 vs. Age	0.086	0.577
IL18 vs. Age at onset	0.088	0.566
IL18 vs. Duration	0.131	0.390
IL18 vs. EDSS	0.065	0.672
sIL2Rα vs. Age	−0.041	0.790
sIL2Rα vs. Age at onset	0.072	0.636
sIL2Rα vs. Duration	−0.094	0.539
sIL2Rα vs. EDSS	−0.071	0.645
sIL7Rα vs. Age	−0.324	0.030
sIL7Rα vs. Age at onset	−0.247	0.102
sIL-7Rα vs. Duration	−0.137	0.370
sIL7Rα vs. EDSS	−0.157	0.302
TNFα vs. Age	−0.061	0.689
TNFα vs. Age at onset	−0.010	0.515
TNFα vs. Duration	0.049	0.748
TNFα vs. EDSS	0.188	0.216

r = correlation coefficient; IGF-1 = Insulin-like growth factor; IL = Interleukin; TNFα = Tumor necrosis factor; EDSS = Expanded disability status scale.

## Data Availability

Data can be assessed upon request to the corresponding author.

## Abbreviations

The following abbreviations are used in this manuscript:

MS	Multiple Sclerosis
IL	Interleukins
IGF	Insulin-Like Growth Factor
TNF	Tumor Necrosis Factor
EDSS	Expanded Disability Status Score
sILR	Soluble Interleukin receptor
AUC	Area Under Curve
ROC	Receiver Operating Characteristics
